# Matrix metalloproteinase-7 and matrix metalloproteinase-9 are associated with unfavourable prognosis in superficial oesophageal cancer

**DOI:** 10.1038/sj.bjc.6601372

**Published:** 2003-11-25

**Authors:** Y Tanioka, T Yoshida, T Yagawa, Y Saiki, S Takeo, T Harada, T Okazawa, H Yanai, K Okita

**Affiliations:** 1 Department of Gastroenterology and Hepatology, Yamaguchi University School of Medicine, Yamaguchi, Japan

**Keywords:** superficial oesophageal cancer, matrix metalloproteinase-7, matrix metalloproteinase-9, lymph node metastasis, prognostic factor

## Abstract

If oesophageal carcinoma is detected in the superficial stage, the prognosis is better than for advanced oesophageal carcinoma. But the factors which predict the prognosis and treatment policy remain unclear. Matrix metalloproteinase-7 (MMP-7) and matrix metalloproteinase-9 (MMP-9) have been reported to have close associations with tumour invasion and metastasis. In this study, we retrospectively studied the relations between MMP-7 and MMP-9 expression in immunohistochemistry, clinicopathologic factors, and prognosis in 55 superficial oesophageal carcinomas. MMP-7 and MMP-9 expression occurred in 23.6% and 47.3% of the patients, respectively. MMP-7 expression was significantly correlated with the presence of nodal metastasis (*P*=0.004). MMP-9 expression was significantly correlated with the depth of tumour invasion (*P*=0.004), lymphatic permeation (*P*=0.001), nodal metastasis (*P*=0.049), and pathologic differentiation grade (*P*=0.003). By the log-rank test, MMP-7 expression and MMP-9 expression on the invasive front were related to the prognosis. In multivariate analysis, MMP-9 expression on the invasive front was an independent prognostic indicator. The combined expression of MMP-7 and MMP-9 may be a good marker for the degree of malignancy of oesophageal cancer and for the presence of lymphatic metastasis.

Oesophageal cancer is a disease with a high degree of malignancy, and its prognosis is worse than that of other gastrointestinal tumours. The two-year survival rate of patients who receive surgery for oesophageal cancer is unfavourable, being about 30% (Krevsky, 1995; Medical Research Council Oesophageal Cancer Working Party, 2002). For those with risk factors for oesophageal cancer, which include smoking and drinking, high age (50 to 70 years), and head and neck carcinomas, a common form of screening is oesophageal endoscopy with iodine solution for detection of oesophageal carcinoma in the early stage. As a result, the detected number of superficial oesophageal carcinomas, in which tumour invasion is limited to the mucosa or submucosa, with a favourable prognosis has increased (Brodmerkel, 1971; Endo *et al*, 1986). With endoscopic ultrasonography (EUS) and endoscopic mucosal resection (EMR) we positively staged and treated oesophageal cancer (Yanai *et al* 1996; Yanai *et al*, 1997; Takeo *et al*, 2001).

Although methods to diagnose superficial oesophageal cancer have advanced, an important reason for the poor prognosis of oesophageal cancer is lymph node metastasis, even in superficial carcinoma. Thus, the existence of lymph node metastasis determines the strategy for treatment and the estimated prognosis of oesophageal cancer. Oesophageal cancer limited to the epithelium (EP) and lamina propria mucosae (LPM) has little lymph node metastasis. But the likelihood of lymph node metastasis for cancer with muscularis mucosae (MM) invasion is almost 10%. The possibility of lymph node metastasis with invasion of the submucosa (SM) increases to as much as 30% to 50% (Bogomoletz *et al*, 1989; Kodama *et al*, 1998). Thus, for tumours with MM and SM invasion, operation is considered necessary even in the case of superficial oesophageal tumours. Thus, new parameters for lymph node metastasis and prognosis are needed for tumours with MM and SM invasion.

Recently, the matrix metalloproteinase (MMP) family has been reported to play important roles in the invasion and metastasis of colon cancer and gastric carcinoma (McDonnell *et al*, 1991; Wilson *et al*, 1997; Adachi *et al*, 1998; Yamashita *et al*, 1998). In particular, MMP-7 is characterized by (1) broad and strong proteolytic activity against a variety of extracellular matrix substrates such as collagens, proteoglycans, laminin, fibronectin, and casein, (2) lack of a c-terminal domain, so it is not regulated easily by tissue inhibitor of metalloproteinases (TIMP), and (3) production by tumour cells themselves (Miyazaki *et al*, 1990; Matrisian, 1992; Baragi *et al*, 1994; Adachi *et al*, 1998). It has been reported that MMP-7 is present in gastric, colorectal, head and neck, and hepatocellular cancers (Yoshimoto *et al*, 1993; Yamamoto *et al*, 1997; Adachi *et al*, 2001; Liu *et al*, 2002). MMP-7 has attracted much attention because it is reported to be present in the tumour invasive front (Adachi *et al*, 1998; Adachi *et al*, 2001; Lin *et al*, 2002). It has also been reported to have a correlation with invasion and metastasis in oesophageal cancer (Adachi *et al*, 1998; Yamamoto, 1999; Ohashi, 2000; Yamashita, 2000).

On the other hand, the members of the MMP family form a cascade and activate each other. MMP-9, which works directly to degrade the extracellular matrix (Parsons *et al*, 1997), is the last stage in the cascade. It decomposes type IV collagen, which is the chief component of basement membrane, and also promotes tumour invasion and metastasis. MMP-9 expression in tumours was reported in gastric, colon, pancreatic, and cervical cancers (Torii *et al*, 1998; Baker *et al*, 2002; Kato *et al*, 2002; Nagakawa *et al*, 2002).

To determine the appropriate treatment for superficial oesophageal carcinoma, we retrospectively studied expression of MMP-7 and MMP-9 and investigated clinical pathological factors and outcomes in superficial oesophageal cancer treated by endoscopic and surgical resection.

## MATERIALS AND METHODS

### Patients and tissue samples

Fifty-five patients (average age 65. 2 years old, male/female ratio 41/14), consisting of 34 with surgical resection for superficial oesophageal cancers between 1995 and 1999, and 21 with EMR between 1995 and 2001 at Yamaguchi University Hospital were studied. No patient had received irradiation treatment or chemotherapy before the resection. Forty-four oesophageal squamous cell carcinomas (SCC) and 1 adenocarcinoma were included. The age range for the 55 patients was 44–85 years (mean, 65.2 years). Lymph node metastases were detected in twelve patients but none of the patients had distant metastasis. The depth of superficial oesophageal tumour invasion was EP in 9 cases, LPM in 6 cases, MM in 16 cases and SM in 24 cases. All 55 patients were followed up. The median follow-up period after the first treatment was 34.2 months (1–81 months), at which time 15 patients had already died. The reasons for death were as follows: oesophageal carcinoma for 5, (survival time 9–47 months, average 22.8 months), other carcinomas for 3, and cerebral infarction for 3.

One of the deepest sections from each tumour was selected for evaluation. It was pathologically classified by standard methods using hematoxylin and eosin (HE) staining as follows (Japanese Society for Esophageal Disease, 2001). According to the depth of invasion, we classified the tumours into 4 groups: EP, LPM, MM, and SM. The tumours were classified as well differentiated SCC, moderately differentiated SCC, poorly differentiated SCC, and others. We also examined whether lymphatic permeation, venous invasion, and nodal metastasis were present. Gross features of the superficial oesophageal cancer were classified as elevated, flat and depressed. Infiltrative growth patterns (inf) were classified into three types: *α* (expansile type: expansile growth of tumour nests forming a distinct border with surrounding tissue), *β* (intermediate type: intermediate pattern, between inf-*α* and inf-*γ*) and *γ* (infiltrative type: infiltrative growth of tumour nests forming an indistinct border with surrounding tissue).

Immunohistochemistry for MMP-7 and MMP-9 was performed for each case. The patient's prognosis after treatment was investigated by examining the medical record. Written informed consent was obtained from all the patients or their relatives.

### Immunohistochemistry

Four-micrometer sections of paraffin-embedded specimens from the deepest parts of the tumours were dewaxed in xylene, and rehydrated through a series of graded ethanol. After heating in a microwave oven (500 watts) for 6 min, the sections were allowed to cool to room temperature in cold water. Endogenous peroxidase was blocked with 3% hydrogen peroxide in methanol for 30 min. Sections were washed twice for 3 min in phosphate–buffered saline (PBS). Primary mouse monoclonal anti-human MMP-7, purified IgG (141-7B2; Fuji Chemical, Toyama, Japan, diluted 1 : 100) and anti-human MMP-9, and purified IgG (56-2A4; First Fine Chemical, Toyama, Japan, diluted 1 : 100) were added and incubated in a moist chamber overnight at 4°C. After three washes in PBS for 5 min each time, samples were incubated with Histofine simple stain MAX-PO (M)(Nichirei, Tokyo) for 30 min at room temperature, according to the manufacturer's instructions. The reactions were stopped by washing the sections three times for 5 min in PBS. Positive reactions were visualized with hydrogen peroxide containing 0.05% 3,3′-diaminobenzine (DAB) in PBS. The slides were counterstained with Mayer's hematoxylin solution for nuclei. Negative control sections were made by omitting the primary antibody.

The score was calculated as the percentage of cells stained positively among tumour cells counted. Four high-power fields (× 400) were selected. Cases were considered positive when over 10% of carcinoma cells were stained. Immunostaining signals at the invasive front were also calculated and the cases were considered positive when not less than 10% of cells at the invasive front were stained (Kawano *et al*, 1997; Yamamoto *et al*, 1999). All specimens were evaluated by two investigators (Y.T., T.Y.) who were blinded to the patients’ clinical information. Disagreements were reviewed and a conclusive judgment was made.

### Statistical analysis

Statistically significant differences were determined by Fisher's exact probability test for gender, lymphatic permeation, venous invasion, and nodal metastasis. Pearson's chi-squared test was performed for gross feature classification, histological differentiation grade, and infiltrative growth pattern. Other factors such as age and depth of invasion were subjected to the Cochran-Armitage trend test. The *P*-value was calculated using exact methods.

Probability distributions of overall survival were calculated using the Kaplan-Meier method and compared by the log-rank test. To determine independent factors that were significantly related to the prognosis for patients with oesophageal cancer, multivariate analysis was performed using Cox's proportional hazards model with a stepwise procedure. The variables included in the multivariate analysis were the following categories: gender, age (under 59, 60–64, 65–69, and over 70), gross features (elevated, flat, depression), depth of invasion (EP, LPM, MM, SM), histological differentiation grade (well differentiated SCC, moderately differentiated SCC, poorly differentiated SCC, and others), lymphatic permeation (positive, negative), venous invasion (positive, negative), infiltrative growth patterns (*α*,*β*,*γ*), nodal metastasis (positive, negative), MMP-7 (positive, negative), MMP-7 on the invasive front (positive, negative), MMP-9 (positive, negative), and MMP-9 on the invasive front (positive, negative). For all statistical tests, a P value of less than 0.05 was defined as statistically significant. All analyses were performed using SAS version 6.11 (SAS Institute Inc., Cray, NC, USA).

## RESULTS

### Expression of MMP-7

**Figure 1:**
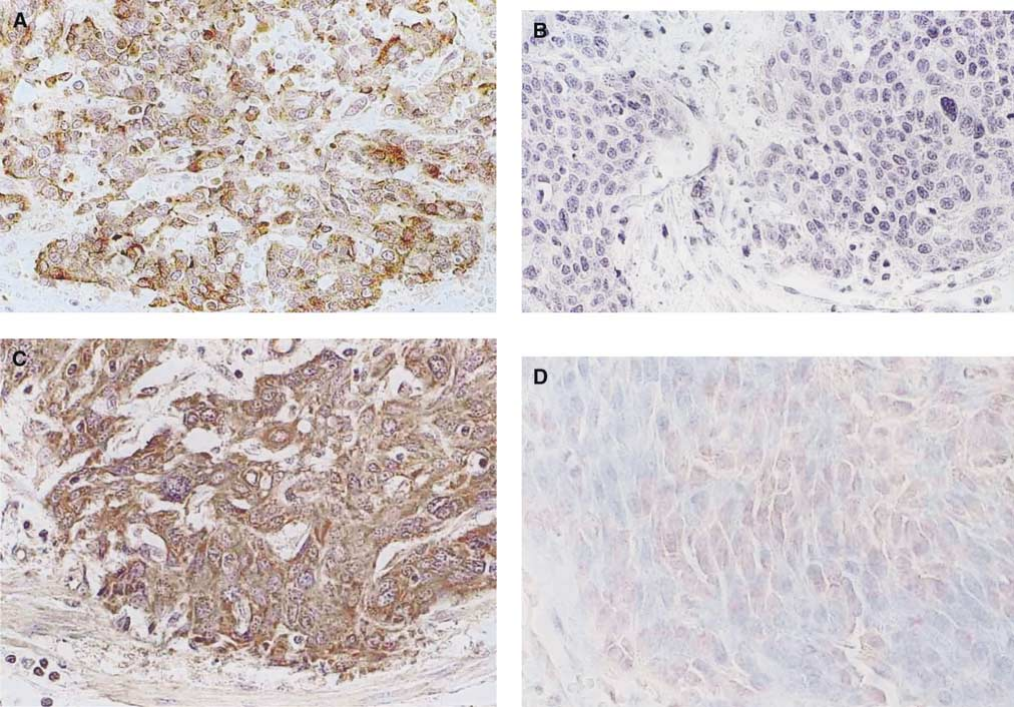
Immunohistochemical staining of oesophageal cancer for MMP-7 and MMP-9. (**A**) MMP-7-positive SCC; MMP-7 was stained in cancer cell cytoplasm, (**B**) MMP-7-negative SCC, (**C**) MMP-9-positive SCC; MMP-9 was stained in tumour cytoplasm, (**D**) MMP-9-negative SCC (× 400).

The cytoplasms and cell membranes of carcinoma cells were stained brown for MMP-7, but stromal cells, except for some monocytes, were not stained (Figure 1A, B

). Expression of MMP-7 was demonstrated in 13 of the 55 cases (23.6%) (Table 1

**Table 1:** Association of MMP-7 expression and clinicopathologic findings

	**MMP-7**			**MMP-7 at invasive front**		
	**Positive**	**Negative**	***P***	**Positive**	**Negative**	***P***
**Variables**	**(*n*=13)**	**(*n*=42)**	**value**	**(*n*=7)**	**(*n*=48)**	**value**
Gender						
Male	11	30	0.447	6	35	0.664
Female	2	12		1	13	
Age						
≦59	2	14	0.461	1	15	0.851
60–64	4	5		3	6	
65–69	3	7		2	8	
70≦	4	16		1	19	
Gross features						
Elevated	4	7	0.291	3	8	0.057
Flat	1	1		1	1	
Depressed	8	34		3	39	
Depth of invasion						
EP	0	9	0.06	0	9	0.013*
LPM	2	4		0	6	
MM	2	14		0	16	
SM	9	15		7	17	
Differentiation grade						
SCC (well)	2	8	0.569	0	10	0.334
SCC (moderately)	9	22		6	25	
SCC (poorly)	1	10		1	10	
Others	1	2		0	3	
Lymphatic permeation						
Positive	8	13	0.059	6	15	0.01*
Negative	5	29		1	33	
Venous invasion						
Positive	1	7	0.664	1	7	1
Negative	12	35		6	41	
Infiltrative growth pattern						
*α*	4	24	0.186	1	27	0.043*
*β*	8	17		5	20	
*γ*	1	1		1	1	
Nodal metastasis						
Positive	7	5	0.004*	5	7	0.004*
Negative	6	37		2	41	

* Significant.

). MMP-7 was immunostained at the invasive front in 7 cases (12.7%). MMP-7 expression was significantly correlated with positive nodal metastasis (*P*=0.004) only. In the invasive front, MMP-7 expression was significantly correlated with deep tumour invasion (*P*=0.013), positive lymphatic permeation (*P*=0.010), positive nodal metastasis (*P*=0.004) and unclear infiltrative growth pattern (*P*=0.043).

### Expression of MMP-9

Expression of MMP-9 was observed in the cytoplasm of carcinoma cells, stromal fibroblasts and vascular endothelial cells (Figure 1C, D). It was detected in 26 cases (47.3%) (Table 2

**Table 2:** Association of MMP-9 expression and clinicopathologic findings

	**MMP-9**			**MMP-9 at invasive front**		
	**Positive**	**Negative**	***P***	**Positive**	**Negative**	***P***
**Variables**	**(*n*=26)**	**(*n*=29)**	**value**	**(*n*=22)**	**(*n*=33)**	**value**
Gender						
Male	22	19	0.13	18	23	0.361
Female	4	10		4	10	
Age						
≦59	7	9	0.562	5	11	0.95
60–64	8	1		7	2	
65–69	5	5		4	6	
70≦	6	14		6	14	
Gross features						
Elevated	6	5	0.867	5	6	0.871
Flat	1	1		1	1	
Depressed	19	23		16	26	
Depth of invasion						
EP	1	8	0.004*	0	9	0.001*
LPM	2	4		2	4	
MM	7	9		5	11	
SM	16	8		15	9	
Differentiation grade						
SCC (well)	2	8	0.003*	2	8	0.32
SCC (moderately)	12	19		12	19	
SCC (poorly)	10	1		6	5	
Others	2	1		2	1	
Lymphatic permeation						
Positive	16	5	0.001*	13	8	0.012*
Negative	10	24		9	25	
Venous invasion						
Positive	5	3	0.455	5	3	0.244
Negative	21	26		17	30	
Infiltrative growth pattern						
*α*	11	17	0.633	8	20	0.298
*β*	14	11		13	12	
*γ*	1	1		1	1	
Nodal metastasis						
Positive	9	3	0.049*	8	4	0.047*
Negative	17	26		14	29	

* Significant.

). At the invasive front MMP-9 was observed in 22 cases (40%). Tumours with MM and SM invasion had significantly more MMP-9 expression (*P*=0.004). Poorly differentiated SCC had more MMP-9 expression than moderately and well differentiated carcinomas (*P*=0.003). MMP-9 expression was significantly correlated with positive lymphatic permeation (*P*=0.001), and positive nodal metastasis (*P*=0.049). On the other hand, expression of MMP-9 at the invasive front was significantly correlated with deep tumour invasion (*P*=0.001), positive lymphatic permeation (*P*=0.012), and positive nodal metastasis (*P*=0.047).

### Coexpression of MMP-7 and MMP-9

We divided subjects into two groups with coexpression of MMP-7 and MMP-9, and the others. Coexpression was detected in 11 cases (20%) (Table 3

**Table 3:** Association of MMP-7+MMP-9 coexpression and clinicopathologic findings

	**MMP-7+MMP-9**			**Invasive front MMP-7+MMP-9**		
	**Positive**	**Others**	***P***	**Positive**	**Others**	***P***
**Variables**	**(*n*=11)**	**(*n*=44)**	**value**	**(*n*=6)**	**(*n*=49)**	**value**
Gender						
Male	10	31	0.255	5	36	1
Female	1	13		1	13	
Age						
≦59	1	15	0.341	1	15	0.863
60–64	4	5		2	7	
65–69	3	7		2	8	
70≦	3	17		1	19	
Gross features						
Elevated	4	7	0.158	2	9	0.134
Flat	1	1		1	1	
Depressed	6	36		3	39	
Depth of invasion						
EP	0	9	0.008*	0	9	0.025*
LPM	0	6		0	6	
MM	2	14		0	16	
SM	9	15		6	18	
Differentiation grade						
SCC (well)	1	9	0.452	0	10	0.132
SCC (moderately)	8	23		6	25	
SCC (poorly)	1	10		0	11	
Others	1	2		0	3	
Lymphatic permeation						
Positive	7	14	0.082	5	16	0.026*
Negative	4	30		1	33	
Venous invasion						
Positive	1	7	1	1	7	1
Negative	10	37		5	42	
Infiltrative growth pattern						
*α*	2	26	0.058	1	27	0.064
*β*	8	17		4	21	
*γ*	1	1		1	1	
Nodal metastasis						
Positive	6	6	0.008*	4	8	0.017*
Negative	5	38		2	41	

* significant.

). MMP-7 expression alone was found in 2 cases (4%), and MMP-9 expression alone in 15 cases (27%). Twenty-seven cases (49%) were negative for both MMP-7 and MMP-9. At the invasive front, the numbers of cases with coexpression, only MMP-7, only MMP-9 and negative expression of MMP-7 and MMP–9 were 6 (11%), 1 (2%), 16 (29%), and 32 (58%), respectively. Coexpression of MMP-7 and MMP-9 was significantly correlated with deep tumour invasion in MM and SM (*P*=0.008), and positive nodal metastasis (*P*=0.008). Moreover, expression at the invasive front was significantly correlated with deep tumour invasion (*P*=0.025), positive lymphatic permeation (*P*=0.026) and positive nodal metastasis (*P*=0.017).

### Survival analysis

**Figure 2:**
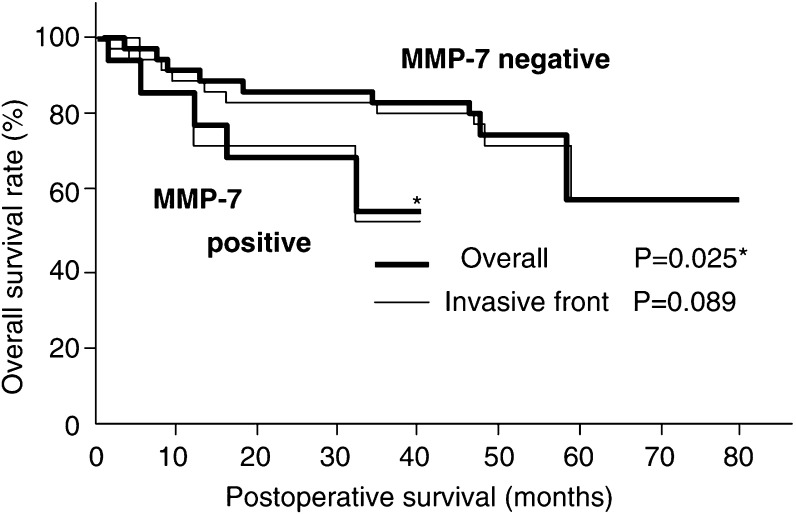
Comparison of overall survival curves for patients with MMP-7-positive and MMP-7-negative superficial oesophageal cancer. MMP-7-positive patients have a less favourable prognosis than those who are MMP-7-negative.

Patients with MMP-7 expression-positive cancer and MMP-9 invasive front-positive cancer had significantly shorter overall survival times than those with negative expression (*P*=0.025, *P*=0.026, respectively) (Figures 2

, 3

**Figure 3:**
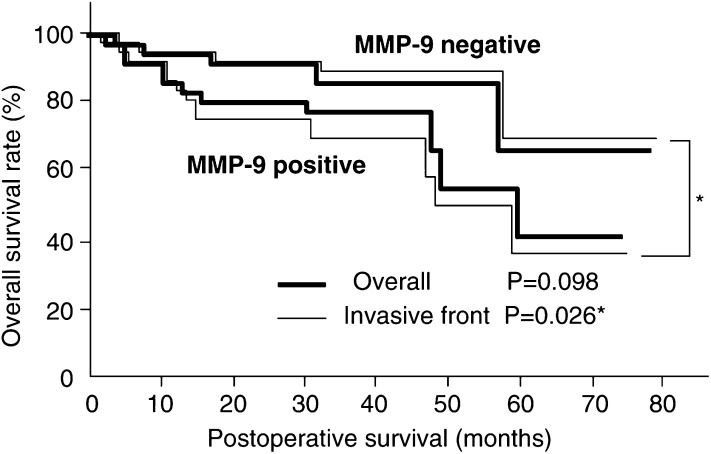
Comparison of overall survival curves for patients with MMP-9-positive and MMP-9-negative superficial oesophageal cancer. Patients who were MMP-9-positive at the invasive front had a less favourable prognosis than those who were MMP-9-negative at the invasive front.

). In the multivariate analysis, only expression of MMP-9 at the invasive front had prognostic significance for overall survival (*P*=0.004, risk ratio 5.68). The other factors were not statistically significant (Table 4

**Table 4:** Multivariate analysis of MMP-7, MMP-9 and pathologic parameters

	**Risk ratio**	**95% CI**	***P* value**
MMP-9 invasive front			
Negative	1		
Positive	5.68	(1.75∼18.444)	0.004*
Age (years)			
≦59	1		
60–64	0.758	(0.102∼5.62)	0.787
65–69	3.652	(0.659∼20.222)	0.138
70≦	4.645	(0.918∼23.491)	0.063

CI=Confidence Interval

* Significant.

). However, older patients tended to have poorer prognoses at the ages of 60–64 (*P*=0.787), 65–69 (*P*=0.138), and over 70 (*P*=0.063).

## DISCUSSION

MMP-7 plays important roles in tumour invasion and metastasis. There have been some reports on MMP-7 in oesophageal cancer. Ohashi *et al*. (2000) examined 148 primary oesophageal cancers immunohistochemically, and showed that cancers with SM invasion had significantly more MMP-7 expression (44%) than those with MM invasion. They also reported that 61.9% of positive venous invasion cases had expression of MMP-7, which was significantly more than in negative cases. Yamamoto *et al*. (1999) used immunohistochemistry to study 100 oesophageal cancers at the invasive front. They reported that the MMP-7 expression rate was 49% and that the expression was related to the depth of invasion (*P*<0.0001), advanced tumour stage (*P*=0.0159), recurrence (*P*=0.0002), recurrence within the first operative year (*P*=0.002), disease-free time (*P*=0.0007) and overall survival (*P*=0.0004). Yamashita *et al*. (2000) reported that the MMP-7 m-RNA expression rate detected by northern blot analysis was 65% in 48 cases, and that expression was related to nodal metastasis (*P*<0.05) and prognosis (*P*<0.01). It was also determined to be of prognostic significance for predicting overall survival (*P*=0.0005) in multivariate analysis.

In our study, MMP-7 expression was significantly related to nodal metastasis (*P*=0.004) and a less favourable prognosis (*P*=0.025). Thus, examination for the presence of MMP-7 in tumour biopsy specimens before treatment is expected to be useful for decision making about the treatment course and to predict prognosis. There was no correlation with age, gender, gross features, deep growth invasion, differentiation grade, lymphatic permeation, venous invasion, or infiltrative growth pattern. The rate of MMP-7 expression was a little low, 23.6% of 55 patients, because we examined only superficial oesophageal cancer.

On the other hand MMP-7 expression at the invasive front was also correlated with positive nodal metastasis. Cases with tumour invasion of the SM had significantly more MMP-7 expression at the invasive front. Moreover, we newly showed that MMP-7 expression at the invasive front was significantly correlated with positive lymphatic permeation and unclear infiltrative growth pattern. These results supported the possibility of an effect of MMP-7 on the tumour invasive front. At the invasive front of oesophageal cancer, MMP-7 may have an important role in invasion and metastasis.

There have been reports about the roles of MMP-9 expression in tumour invasion and metastasis, but this is controversial in oesophageal cancer. Ohashi *et al*. (2000) immunohistochemically studied 148 oesophageal cancers, and found that tumours with SM invasion had higher MMP-9 expression (51.9%) than those with MM invasion. They showed that the presence of venous invasion was related to MMP-9 expression, as it occurred in 65.1% of cases. But Koyama *et al*. (2000) found 60% expression in immunohistochemistry for 39 oesophageal tumours, and reported that there was no relation of MMP-9 expression to any clinicopathologic factor. They found a relation of MMP-9 expression to venous invasion (*P*=0.0022) by zymography, so active MMP-9 protein might lead to tumour invasion. Sato *et al*. (1999) found no relation to depth of invasion, nodal metastasis, distant metastasis, staging, sex, age or differentiation grade. Murray *et al*. (1998) also reported that MMP-9 expression had no relation to staging.

In our study, MMP-9 expression at the invasive front was significantly related to deep invasion, positive lymphatic permeation, and positive nodal metastasis, like MMP-7 expression at the invasive front. In the MMP family activation cascade, MMP-9 is downstream of MMP-7 and destroys the extracellular matrix directly (Parsons *et al*, 1997). Judging from the above, it is reasonable to consider that MMP-9 expression behaves similarly to MMP-7 expression. Moreover, in both univariate and multivariate analyses, MMP-9 expression at the invasive front was an independent prognostic factor. Thus MMP-9 expression at the invasive front in the resected tumour may indicate the necessity for additional treatment after operation.

MMP-9 expression for the whole tumour was also related to the oesophageal tumour differentiation grade, along with depth of invasion, lymphatic permeation and nodal metastasis. MMP-9 may have a role in the destruction of the extracellular matrix of the tumour overall, not only at the invasive front. Thus, MMP-9 expression examined by a pre-treatment biopsy of the tumour surface may be a good marker for lymphatic permeation, nodal metastasis and depth of invasion. The presence of MMP-9 expression may be important to determine the strategy for treatment.

We examined the significance of MMP-7 and MMP-9 coexpression and classified patients into two groups, those with both MMP-7-positive and MMP-9-positive expression and the others. Coexpression was significantly correlated with deep invasion into the MM and SM, and nodal metastasis. Although it had no relation with minute changes like lymphatic permeation, coexpression of MMP-7 and MMP-9 may suggest nodal metastasis in oesophageal cancer.

Even superficial oesophageal cancers with invasive depths of MM and SM have about 10–50% lymph node metastasis, so it is often difficult to choose between EMR and surgical resection. Immunohistochemistry for MMP-7 and MMP-9 before treatment in these cases will provide important information for treatment policy. MMP-7-negative and MMP-9-negative biopsy findings might be useful as an indication for EMR without operation because they could predict shallow depth of invasion and little lymphatic metastasis.

In conclusion, combined MMP-7 and MMP-9 expression may be a good marker for the malignancy level of oesophageal cancer and for the presence of lymphatic metastasis, not venous invasion. MMP-9 expression at the invasive front can be a significant prognostic variable for predicting overall survival.
